# Should we get involved? impact of human collaboration and intervention on multi-robot teams

**DOI:** 10.3389/frobt.2025.1526287

**Published:** 2025-10-15

**Authors:** Joseph Bolarinwa, Manuel Giuliani, Paul Bremner

**Affiliations:** 1 Bristol Robotics Laboratory, University of the West of England, Bristol, United Kingdom; 2 Manchester Centre for Robotics and AI, Department of Computer Science, The University of Manchester, Manchester, United Kingdom; 3 Kempten University of Applied Sciences, Kempten, Germany

**Keywords:** multi-agent systems, simulation, centralised architecture, decentralised architecture, Human collaboration, human intervention, human-in-the-loop, multi-robot teams

## Abstract

**Introduction:**

The challenges encountered in the design of multi-robot teams (MRT) highlight the need for different levels of human involvement, creating human-in-the-loop multi-robot teams. By integrating human cognitive abilities with the functionalities of the robots in the MRT, we can enhance overall system performance. Designing such a human-in-the-loop MRT requires several decisions based on the specific context of application. Before implementing these systems in real-world scenarios, it is essential to model and simulate the various components of the MRT to evaluate their impact on performance and the different roles a human operator might play.

**Methods:**

We developed a simulation framework for a human-in-the-loop MRT using the Java Agent DEvelopment framework (JADE) and investigated the effects of different numbers of robots in the MRT, MRT architectures, and levels of human involvement (human collaboration and human intervention) on performance metrics.

**Results:**

Results show that task execution outcomes and request completion times (RCT) improve with an increasing number of robots in the MRT. Human collaboration reduced the RCT, while human intervention increased the RCT, regardless of the number of robots in the MRT. The effect of system architecture was only significant when the number of robots in the MRT was low.

**Discussion:**

This study demonstrates that both the number of robots in a multi-robot team (MRT) and the inclusion of a human in the loop significantly influence system performance. The findings also highlight the value of simulation as a cost- and time-efficiency strategy to evaluate MRT configurations prior to real-world implementation.

## 1 Introduction

Complex problems that exceed the capabilities of a single robot can be addressed by a multi-robot team (MRT) (Darmanin and Bugeja, 2017). The use of MRTs is not limited to using the robots to execute different tasks (Yang and Parasuraman, 2020), but also in scenarios where the robots have similar capabilities and may be used to execute similar concurrent tasks (Chang et al., 2021). Application domains of MRTs include surveillance, search and rescue (Stancovici et al., 2016; Mendonça et al., 2016), foraging and flocking (Parker, 1998; Lein and Vaughan, 2009; Gu and Wang, 2009), formation and exploration (Wang et al., 2016; Phanichnitinon et al., 2014), large scale assembly lines (Simmons et al., 2001) and adversarial or extreme environments (Zhang and Wang, 2007; Agmon et al., 2008).

Due to the technical complexity of MRTs, a variety of technology-related research topics have been explored, these include communication and architectures (Gielis et al., 2022), task allocation (Chakraa et al., 2023), localization (Chen et al., 2025), mapping and exploration (Kwa et al., 2022), manipulation, and motion coordination (Chang et al., 2021). In most of this research, the assumption is that the MRT is programmed to act autonomously without human involvement. However, in real-world deployments of MRTs in many industries a human-in-the-loop approach is required when using MRTs. For example, the safety-conscious nuclear industry does require a human operator to either tele-operate robots directly, or at least monitor their actions.

Human-robot interaction (HRI) has been studied in the field of MRTs (Villani et al., 2020; Patel and Pinciroli, 2020), but most of the previous HRI MRT research have focused more on the human carrying out supervisory activities. In the research presented in this paper, we introduce HRI to MRTs where the human performs roles beyond just monitoring the robots, but also collaborates with the robot. Human-Robot collaboration involves the human and robot working together simultaneously on a shared goal, in the form of physical collaboration or contactless collaborations (Hjorth and Chrysostomou, 2022). The main goal of our work is to evaluate how different levels of human involvement affect the overall performance of an MRT.

In order to evaluate the impact of human involvement on MRTs, we developed and implemented an MRT simulation environment. This has the advantage that we can evaluate a broad set of MRT system parameters and operational contexts in a short time frame. We modelled the MRT as a multi-agent system (MAS) and simulated a set of scenarios. Each scenario comprises a combination of MRT architecture type (centralised, decentralised), human involvement (no involvement, collaboration, intervention), and number of robots (4, 6, 8, 10). We measured the impact on several performance parameters, including request execution outcome (success/failure), number of successful tasks, number of failed tasks, and request completion time.

This work makes two main contributions to the knowledge about human-in-the-loop MRT: (1) we present a simulation framework based on a MAS that simulates a robot team for nuclear decommissioning tasks with a human in the loop, and (2) we evaluated the impact of different levels of involvements in a human in the loop MRT on centralised and decentralised architectures.

## 2 Related work

Research on MRTs and their implementations for different applications reveal the different MRT themes that may be explored. Due to these large number of research themes, this literature review only covers topics relevant to this paper, which include modeling a MRT as a multi-agent system (MAS), and modelling and simulation environments for agent based systems. Modelling an MRT as an MAS allows us to define relevant actors in the MRT as agents, thus making it possible to model the behaviours of, and communication between, all actors in the system. We also reviewed literature on human-in-the-loop multi-agent teams, since it is a theme of interest to this paper, providing an overview of the state of research in this MRT theme. In order to model and simulate our human-in-the-loop MRT as a MAS, we also explored literature on modelling and simulation environments for agent based systems.

### 2.1 Multi-agent systems

An MAS consists of autonomous entities called agents that collaboratively solve complex tasks. Dorri defined an agent as an entity in an environment that has the ability to sense different parameters that may be used to make decisions to achieve the goal of the entity (Dorri et al., 2018). Agents operate by sensing parameters from the environment, using knowledge obtained from neighbouring agents, and using history of previous actions taken (Garcia et al., 2010).

Important features in MAS and the corresponding categories that arise from these features were outlined by Dorri et al. (2018). The features and their corresponding categories include leadership [leader-follower, leaderless (Fu and Wang, 2014; Li et al., 2011)], decision function [linear, non-linear (Zhao et al., 2013; Li et al., 2012)], heterogeneity (heterogeneous, homogeneous (Kim and Matson, 2016; Vrancken and Soares, 2009)), agreement parameters (first order, second order, high order (Miao and Ma, 2015; Wen et al., 2013)), delay consideration [with and without time delay (Gao et al., 2016; Du et al., 2013)], topology [static and dynamic (Liu et al., 2015; Olfati-Saber and Murray, 2004)], data transmission frequency (time triggered, event triggered (Guo et al., 2014; Li et al., 2014)), and mobility [static and mobile agents (Wooldridge, 2009; Wang et al., 2014)].

The properties and collaborative behaviours of agents therefore make them suitable to represent challenges commonly faced in MRT, such as cooperation and coordination between robots, non-deterministic dynamic environments which may increase the complexity of their decision making, and trajectory planning (Ota, 2006; Soriano et al., 2013; Iñigo-Blasco et al., 2012; Duan et al., 2012). With these features in mind, we modelled our MRT as MAS that includes a decision and task allocation system, and multiple leaderless homogeneous robots. To the best of our knowledge, no prior work has been carried out in modelling a human-in-the-loop MRT as a MAS for investigative research,where the effect of different levels of human involvement is explored for different MRT architectures.

### 2.2 Human-in-the-loop multi-robot teams

Although there are advances in technologies that improve the autonomous capabilities of robots employed to carry out tasks, sometimes application areas, such as nuclear decommissioning, insist on having a human in the loop. Whilst there are several reasons why there may be the need to have a human in the loop, such as the safety case in nuclear decommissioning, one advantage is that it allows for the integration of robots and human capabilities. Hence, it makes it possible to integrate the superior capabilities of the robot, in terms of precision and being able to operate in dangerous environments, with human cognitive capacities (Ajoudani et al., 2017). Likewise, as the environment becomes more unpredictable, unexpected problems may be solved with human input, e.g., monitoring, fault detection, and recovery (Kaufmann et al., 2021).

Introducing a human into an MRT however has its challenges. The complexity of task coordination varies with scenario, and as the number of robots in the MRT increases, a greater level of multitasking may be required. Therefore, as operators switch between tasks, the chance of human error and of human-induced complications increases. In general, performance of an MRT with a human in the loop may be affected by the flexibility of task allocation (Valenchon et al., 2022), the structure of the MRT (Gao et al., 2014), factors that may affect supervisory control like the number of concurrent tasks (Cummings et al., 2010; McKendrick et al., 2014) and human-induced errors. It is therefore important to find a balance in assigning responsibilities to the different components in the system based on known information about the limitations in their capabilities. A possible solution is to simulate a human-in-the-loop MRT to understand how roles assigned to the human and MRT may affect the success of executing requests before implementation for real-world applications.

### 2.3 Simulated MultiRobot teams applications

One of the challenges of integrating a human into a Multi-Robot Team (MRT) is scalability. While scalability in MRTs is often advantageous, allowing for flexible adaptation to varying job sizes and dynamic demand levels, it is constrained by human cognitive limitations and decision-making speed (Humann and Pollard, 2019) examined the impact of humans on the scalability of multirobot systems and simulated the challenges of scaling a multi-operator, multirobot surveillance system. Their review highlighted several human-related factors that limit scalability, including reduced situational awareness, errors due to high workload, and declining precision in control inputs. Using an agent-based model developed with the open-source software GAMA, the surveillance simulation incorporated human operator agents, quadrotor UAVs, and fixed-wing UAVs. The results indicated that a single operator could effectively control up to three robots, and increasing the number of controllable robots required adding more human operators to the system.

Al-Hussaini et al. (2021) developed adaptive techniques using Monte Carlo forward simulations to predict future mission states by constructing probability distributions of potential outcomes in complex environments, thereby enabling alert generation. This approach allowed for accurate real-time alert generation in scenarios where computational time is limited to just a few seconds.

To assess proposed team designs in uncertain Military Operations in Urban Terrain (MOUT) scenarios and identify the most critical design factors influencing team performance (Giachetti et al., 2013), developed a simulation model incorporating team coordination and human-robot interaction. The findings indicate that larger teams outperformed the effects of noise factors such as danger level and robot reliability, with robot reliability being a key determinant in human-robot team formation. Additionally, the results suggest that as team size increases, centralized decision-making may lead to communication challenges.

In one of the agility challenges during the “Agile Robotics for Industrial Automation Competitions” (ARIAC) 2023, the human operator was modeled as a “Belief-Desire-Intention (BDI)” agent using Jason. Participants were required to control a gantry robot, four automated guided vehicles, and various other components to navigate agility challenges within a simulated factory environment using ROS 2 (Robot Operating System) and Gazebo (Becker et al., 2023). Different behavioral models were implemented to define how the human operator responded when near the robot, ranging from minimally intrusive to highly intrusive interactions. Additional simulation studies have been conducted by Maoudj et al. (2015), Carlin et al. (2010), Mota et al. (2011), Zhang et al. (2012), Dawson et al. (2010), Harbin et al. (2021), Humann et al. (2023), An et al. (2023), Street et al. (2023).

The reviewed MRT simulation applications demonstrate that multirobot teams have been successfully simulated across various application contexts. However, the extent to which the behaviors of MRT components can be defined and simulated varies, as some environments function as black boxes. Additionally, none of the reviewed studies explored MRT simulations in a nuclear context. Consequently, our selection of a simulation environment prioritized the ability to precisely define the behaviors of MRT components.

### 2.4 Modelling and simulation environments for agent-based systems

There are several performance metrics of agent-based systems that may be defined, analysed and evaluated using different methods. However, the choice of methods may vary depending on the MRT application, goal of the system, or the agent-based system. Some of the commonly used multi-agent frameworks include the Java Agent DEvelopment framework (JADE) (Sadik et al., 2019; Bellifemine et al., 2007), GAMA (GAMA, 2023), Matlab (Panasetsky and Tomin, 2013), Repast (North et al., 2006), MASON (Luke et al., 2005), Netlogo (Tisue and Wilensky, 2004), and Anylogic (Borshchev, 2014).

For the evaluation reported in this paper, we employed the JADE to model, simulate, and evaluate different scenarios of an MRT. JADE was chosen because it allows the creation of agents and redefinition of behaviours by providing relevant class libraries. Hence, it makes it possible to define in detail the attributes and behaviours of each components of the MRT. It is also easier to translate Business Process Model and Notation (BPMN) activity diagrams, which were used to model simulation framework components, into JADE agents.

## 3 Materials and methods

A human-in-the-loop MRT consists of a human operator and different components of the MRT. In this section we provide a detailed description of a simulation framework model to evaluate human involvement in an MRT. In this paper, we have explored the context of nuclear decommissioning as the application area; however, the results and recommendations may also be applicable to other MRT contexts. Components of the system were modelled using the BPMN (Camunda, 2025), an architecture description language that extends the Unified Modelling Language (UML) (Object Management Group, 2025; Sadik et al., 2019). BPMN is capable of describing logical flows of activities within a process and provides a rich set of notations, each having a predefined semantic. The BPMN notations that have been used to construct analytical models of the different MRT architecture use cases investigated in this paper are described in Supplementary Figure 1. The notations have been added to facilitate the interpretation of the various activity diagrams, which illustrate the flow of information across the use cases. Explanations for each activity diagram are also provided. The simulation framework is discussed in Section 3.1. We describe the characteristics of the centralised and decentralised architectures as modelled in this paper in Sections 3.2, 3.3, respectively. These sections also describe how the different components vary between different architectures. The different use cases of varying levels of human involvements are discussed in Section 3.4.

### 3.1 Simulation framework

**FIGURE 1:**
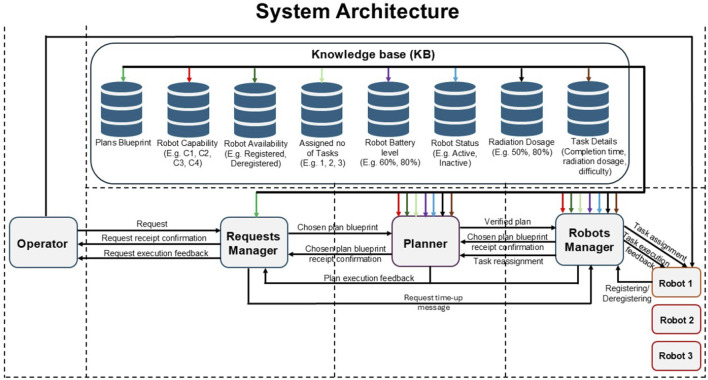
Overview of inter-agent information flow in the system design.

We implemented a framework that simulates centralised and decentralised MRT. Figure 1 shows an overview of the MRT system architecture. The architecture consists of the following components: Operator, sends out requests to MRT for tasks that are to be executed; Requests Manager, identifies relevant plan blueprints that may be used to execute requests; Planner, develops a verified plan for a request; Robots Manager, assigns tasks to robots and handles registering and deregistering of each robot; Robot, executes tasks assigned to it based on its capabilities; and the Knowledge Base (KB), a central repository for keeping track of plan blueprints, robots, and tasks. The Knowledge Base is implemented as catalogues in code. Figure 1 also shows the information flow between components.

The simulation framework has been implemented using JADE (Jade, 2023). It includes a runtime environment where JADE agents can live. It also consists of a library of classes that can be used to develop agents. Each instance of JADE runtime is an independent thread which is made of a set of containers. A group of agents running under the same runtime instance is called a container, and a set of active containers is called a platform. For a platform to be functional, it must contain an active special main container. The main difference between a main container and normal container is that it holds the Agent Management System (AMS) and the Directory Facilitator (DF). Whilst the AMS provides the naming service, ensuring that each agent in the platform has a unique agent identifier (AID), the DF provides a yellow pages service by which an agent can find other agents.

The functionalities of agents are typically carried out within “behaviours”. A behaviour is an event handler routine that is used by the agent to modify its parameters and interact with other agents. Behaviours offered by JADE include Simple behaviour, One-shot behaviour, Cyclic Behaviour, Composite behaviour, Sequential Behaviour, Parallel behaviour, Finite State Machine behaviour, Waker behaviour, and Ticker behaviour. The behaviours employed for the implementation of our simulation framework include the following:

• One-shot behaviour: this is a simple behaviour that is executed once when it is called by the agent. It is often used to trigger an event and send an ACL-Message.
• Cyclic behaviour: this is a simple behaviour that stays active as long as the agent is alive.
• Sequential behaviour: this is a composite behaviour that controls the sequence of execution of more than one one-shot behaviour.
• Parallel behaviour: this is a composite behaviour that concurrently controls the execution and termination of more than one one-shot behaviour.

We represent every component in the different architecture scenarios as JADE software agents. The JADE description above informs JADE as a proper tool to implement BPMN models in the context of MRTs. This is particularly relevant because an activity from the BPMN can be coded as a simple one-shot or cyclic behaviour in JADE, while a gateway can be translated into composite or parallel behaviours in JADE. We can also use JADE to simulate the MRT and to examine the different scenarios as used in this paper. Likewise, the same implementation code can be used to deploy the system over real world hardware (Sadik et al., 2017).

It is important to consider different concepts when designing an MRT. One such concept is the group architecture, which provides the infrastructure that determines the capabilities and limitations of the system. Some of the features include centralisation/decentralisation, differentiation (homogeneous and heterogeneous), and communication (via environment, via sensing, and via communications) (Cao et al., 1997). While centralised architectures have a single control agent, decentralised architectures do not. Decentralised architectures may furthermore be divided into two types, namely distributed architectures and hierarchical architectures. In distributed architectures, all agents are equal with respect to control, but hierarchical architectures exhibit local centralisation. In this work, centralised and distributed decentralised architectures have been modelled and simulated. Centralized and decentralized architectures are two of the most common approaches for MRT architectures (Parker, 2008), which led to our decision to explore them further. The main difference between the centralised and decentralised architectures modelled in this paper is access to system information and how task allocation was implemented. The following sections describe our centralised and decentralised MRT architecture designs in more detail.

### 3.2 Centralised multi-robot team architecture

The main feature of the modelled centralised architecture presented in this paper is the single control agent. This agent has knowledge of all robots and their states, and is involved with task allocation for all robots based on access to their information. We briefly describe the different components of the system, modelled as individual agents. In order to allocate tasks in a given request, the chances of failure (minimum = 0, maximum = 1, Equation 4) of all capable Robots are calculated for all tasks. Tasks are therefore allocated starting with the task with the least chance of failure.

**FIGURE 2:**
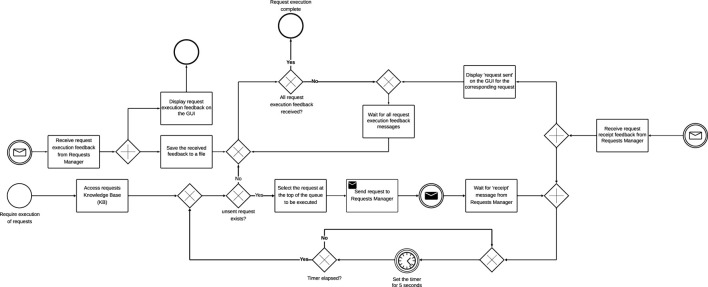
Centralized architecture: operator activity diagram.

Operator: responsible for making requests that are to be executed. Figure 2 shows the activity diagram for the Operator agent. Each request is made up of a set of tasks. All requests to be executed are stored in the Knowledge Base (KB), and on a first-come-first-serve basis, each request in the KB is sent to the Requests Manager in 5 s intervals until all the requests in the KB have been sent. For every request received, the Requests Manager sends a request receipt acknowledgment to the Operator. After each acknowledgement, the Operator updates the status of the request on its graphical user interface (GUI) from ‘waiting’ to ‘request sent’. The GUI is provided so that request executions can be observed in real-time to monitor the system for errors in operation. Simultaneously, the Operator waits for request execution feedback requests from the Requests Manager. After receiving the feedback, the Operator updates its GUI and an Excel file with information on the outcome of the request, the number of successful tasks in the request, the number of failed tasks in the request, the number of times tasks have been reassigned, and the request completion time. The different actions performed by the Operator are executed in separate threads for concurrent execution of actions.

**FIGURE 3:**
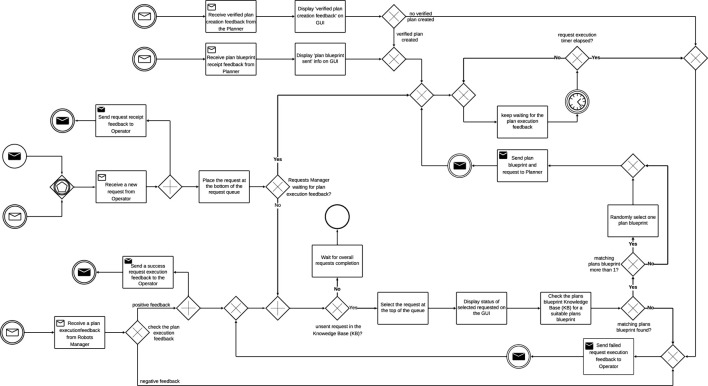
Centralized architecture: Requests Manager activity diagram.

Requests Manager: responsible for identifying the relevant plan blueprint that may be used to execute each request. The different actions performed by the Requests Manager are executed in separate threads for concurrent execution of actions as shown in Figure 3. Each plan blueprint in the KB contains tasks and the capabilities a Robot must possess to complete each task. Requests received from the Operator are stored in the KB and randomly chosen for execution. A timer is set when a request is chosen for execution and if request execution feedback is not received within the set time, the request execution fails. If the Requests Manager finds one or more matching blueprints, a blueprint is randomly selected. The selected plan blueprint and the request are then sent to the Planner to develop a verified plan. The Request Manager also waits for blueprint receipt feedback from the planner. However, if no relevant plan blueprint is found, a “failed request (no blueprint)” message is sent to the Operator. Simultaneously, the Requests Manager also listens for verified plans feedback message from the Planner and requests execution feedback message from the Robots Manager.

Verified plan feedback is received from the Planner if no verified plan may be developed by the Planner. The requests execution feedback message received from the Robots Manager may be positive or negative. The message will be positive if all task executions succeed but negative if the execution of at least one task in the request fails. The request execution feedback message sent to the Operator may also be positive or negative. The message will be positive if all the tasks contained in the request are successfully executed. However, the message will be negative if no verified plan can be developed, if the request is not executed within the required time, or if the execution of at least one of the tasks in the request fails. Simultaneously, the Requests Manager GUI is also updated with the plan blueprints for all the requests and request execution status.

**FIGURE 4:**
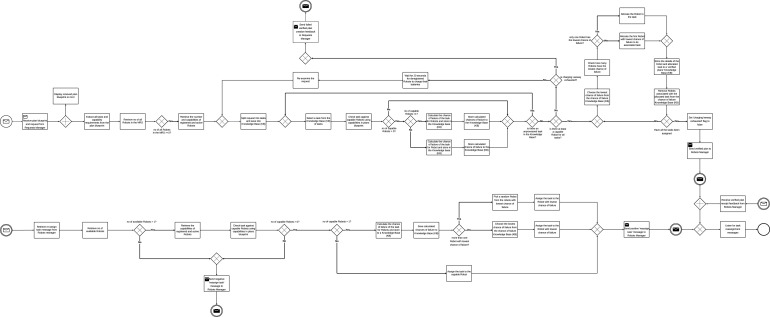
Centralized architecture: Planner activity diagram.

Planner: responsible for developing a verified plan on how to execute a request. The different actions performed by the Planner are executed in separate threads for concurrent execution of actions, shown in Figure 4. In order to develop the verified plan, the Planner splits the request into separate tasks and retrieves the capabilities required to execute the task from the plan blueprint. The Planner also retrieves information on all available Robots, and checks if each Robot has the capabilities required to complete the task. We assume that there must be more than one Robot for consideration to increase the chance of having a capable Robot to assign the task to. Likewise, having more than one capable Robot makes it possible to execute tasks concurrently, hence reducing the request completion time. If only one Robot is available for the request execution, a “failed (Insufficient robots)” message is sent to the Requests Manager for the chosen request. The capable Robots must also have sufficient power (battery life) to complete the task. Battery life is crucial, as, unlike industrial robots or those stationed in fixed locations (Bolarinwa et al., 2019; Boschetti et al., 2021; Bolarinwa et al., 2022), many robotic applications require mobility or deployment in environments where tethered connections to alternating current power sources are not feasible (Baniqued et al., 2024; Azpúrua et al., 2023). A Robot is not considered for a task if its battery life is lower than that required to complete the task, even if the Robot has the capabilities required to execute the task. If no capable Robot is found for a task in the request, a ‘charging leeway’ timer is set. The charging leeway timer is introduced for instances where a capable Robot is not found due to the robot being deregistered for its battery to be charged. The timer is chosen to be longer than the Robot’s charging time. When the timer elapses, the process is repeated to identify capable Robots.

We assume that the centralised architecture has complete information about all the Robots and tasks. Therefore, for all the tasks in the request, the chances of failure of all capable Robots are calculated and stored in a KB. Task allocation then starts with the Robot with the lowest chance of failure. When the Robot with the lowest chance of failure is assigned to a task, all the other robots considered for the allocated task and their calculated chances of failure are removed from the catalogue. If more than one Robot has the lowest chance of failure for a given task, a Robot is randomly chosen. The next lowest chance of failure is selected for the corresponding task and Robot. This process is repeated until all the tasks are allocated to Robots. If at least one of the tasks could not be assigned a Robot, a ‘failed (No sufficient capable Robots)’ message will be sent to the Requests Manager.

Our task allocation algorithm ensures that the robot with the lowest chances of failure is always allocated the task. However, there is a chance that a robot may be allocated more than one task from a request. The allocated tasks and corresponding Robots form the verified plan that is then sent to the Robots Manager. The Planner also waits for a ‘verified plan’ receipt message from the Robots Manager to ensure that the plan is received.

Simultaneously, the Planner receives task reassignment messages from the Robots Manager and stores the messages in the KB. If a previously allocated task fails, and the request execution time has not elapsed, the request is sent back to the planner to reassign the task to another Robot. To reassign the task, if the number of capable Robots is more than one, task allocation is done using the chances of failure of the capable Robots. If more than one capable Robot has the lowest chance of failure, a Robot is randomly chosen and sent to the Robots Manager. If no Robot is found, a ‘no robots found’ message is sent to the Robots Manager. If no capable Robots are found, a ‘failed (No capable robot)’ message is sent to the Robots Manager.

**FIGURE 5:**
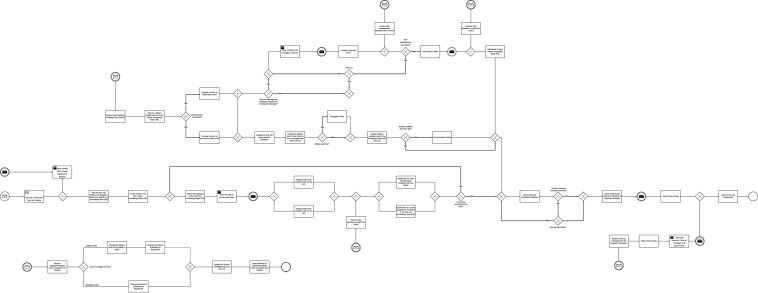
Centralized architecture: Robots Manager activity diagram.

Robots Manager: responsible for assigning tasks to the Robots and also handles registering and deregistering of each Robot. The different actions performed by the Robots Manager are executed in separate threads for concurrent execution of actions, as shown in Figure 5. The Robots Manager manages a KB of the Robots’ information. The information stored in the KB include Robot capabilities, Robot availability, number of tasks assigned to each robot, battery power level of each Robot, radiation exposure dosages of each Robot, status of each Robot. The simulation is designed such that other agents can access and modify the KB when needed.

The Robot capabilities KB contains information about the capabilities of each Robot which makes it possible to assign each Robot to tasks. The availability KB is updated as each Robot registers or deregisters. A Robot may deregister from the team of Robots if it needs to recharge its batteries, and registers when its batteries are fully charged. When a Robot is deregistered, it cannot be assigned to a task. We represent the battery power level with a range of numbers from 0 (batteries depleted) to 100 % (batteries fully charged). Each task is assigned a number which represents the percentage of battery power required to complete the task. This number is subtracted from the Robot’s battery power level as the task is completed. When the battery level drops to 14 % (a value at which no task may be executed in our simulation), the Robot deregisters to recharge its batteries. We represent the charging process by setting a timer for 10 s, after which the Robot’s battery power level is updated to 100 and the robot sends a message to the Robots Manager to register it into the MRT. The charging time of 10 s was chosen after repeated simulations, to be just long enough for ongoing tasks to be completed but not too long as to delay the request execution. In reality, calculations can be made to determine how long it would take to charge the batteries. The charging process may be designed to be fast enough to avoid delays in request executions. As each Robot is assigned a task, the KB of the number of assigned tasks is updated for each Robot. After completing the task, the Robots Manager also updates the Robot’s radiation exposure dosage KB based on the task’s assigned radiation dosage. The minimum radiation dosage of a Robot is set to 0, while the maximum is set to 100. When a Robot’s radiation dosage reaches 100, the Robot is decommissioned and can no longer be assigned a task. Hence the Robot status KB will be updated from “active” to “decommissioned”.

The Robots Manager also manages the task information KB. The information managed include task completion time, task difficulty, and task radiation dosage. The task completing time (TCT) varies for different tasks. This was simulated by creating a timer using a loop. The loop was used instead of a sleep function to make it possible to interrupt the task execution when the request completion time elapses. Task difficulty also varies from task to task as we assigned values to tasks based on our perceived difficulty levels. For example, we assume that a mapping task will have lower task difficulty than a task which requires a robot to stack barrels as stacking up barrels would require more sensing and actuating, as well as greater level of precision. Higher numbers imply higher difficulty. Task radiation exposure dosage also varies based on the task and has been defined numerically as the amount of radiation exposure a robot is exposed to as it carries out a task. Other agents can also access the task specification KB.

The Robots Manager receives the verified plan from the Planner and sends receipt feedback message to the Planner. The Robots Manager splits the verified plan into tasks, assigned Robots, and sends each task to its assigned Robot. The Robots Manager also stores the task and assigned Robots info in a separate KB. As each Robot completes its assigned task, its ID and assigned task are removed from the KB. This makes it possible to keep tabs on busy Robots. If the request completion time elapses before all the tasks are completed, the information in the KB is used to send messages to busy Robots to cancel their tasks and the request fails. The Robots Manager updates its GUI after receiving task receipt feedback message from the Robot.

Simultaneously, the Robots Manager waits to receive “Register/Deregister” messages from any of the Robots. If a Register message is received, the Robots Manager adds the robot to the MRT by changing the availability of the Robot to “Registered” and the battery level to 100. If a ‘deregistered’ message is received, the Robots Manager removes the Robot from the MRT by changing the availability of the Robot to “Deregistered”.

The Robots Manager also listens for task completion messages from Robots. As each Robot completes the assigned task, its busy status is changed from “busy” to “not busy”. The task completion feedback message could show that the task succeeds or fails. When a ‘task failed’ message is received, and the request completion time has not elapsed, the Robots Manager sends the task to the Planner to be assigned to another Robot. If the task fails, and the request completion time has elapsed, the task is not reassigned but the request fails. If a ‘task successful’ message is received, the number of successful tasks is increased and the battery level, as well as the radiation dosage of the robot is updated. Within the request completion time, if the number of successful tasks equals the number of tasks in the request, the request execution completes, and a request execution feedback message is sent to the requests manager. The Robot Manager also updates its GUI as messages are received and actions are executed.

**FIGURE 6:**
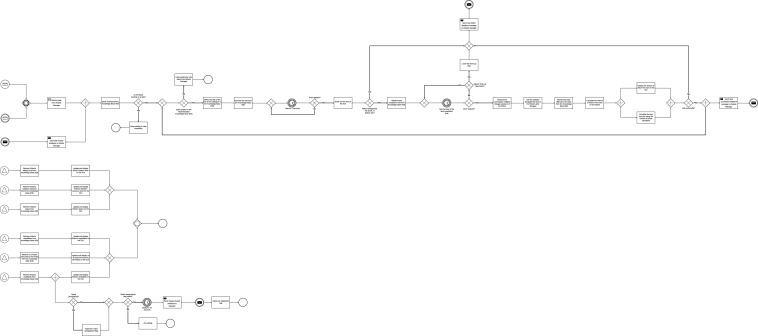
Centralized architecture: Robot activity diagram.

Robot: Each robot in the MRT is simulated using a separate Robot agent. The different actions performed by the Robot are executed in separate threads for concurrent execution of actions, as shown in Figure 6. The Robot is responsible for executing tasks that have been assigned to it based on its capabilities. The Robot receives assigned tasks and sends a receipt message to the Robots Manager. The Robot checks its properties KB and updates its GUI. The GUI makes it possible to monitor processes going on within each agent. The Robot sends messages to the Robots Manager to deregister it from the MRT when its battery level falls below a set value. The Robot receives tasks from the Robots Manager, stores in its internal KB, and randomly selects the tasks for execution.

If a Robot becomes deregistered, inactive, or the battery level drops below a set level before it begins to execute a task, the task fails and a “task failed message” is sent to the Robots Manager. However, if not, the Robot proceeds to execute the task. If the Robot receives a ‘time up’ message as it executes the task, the task execution is cancelled and a “failed” message is sent to the Robots Manager. Tasks execution is simulated using a while loop with 1 s sleep function. The task execution result is simulated by calculating the chance of failure using the task difficulty and cumulative radiation dosage as shown. The chance of failure is then fed into a random number generator. If the number generated is more than the chance of failure, the task succeeds. However, if the number generated is less than the chance of failure, the task fails. This makes it possible to dynamically vary the success or failure of the task based on the task executed and the task execution history of the Robot.

### 3.3 Decentralised architecture

In the decentralised architecture modelled in this paper, tasks are put up for auction and each Robot bids for the task by calculating its chance of failure of the auctioned task. This means that for task allocation, there is no need for the task allocation agents to be aware of the conditions or states of each robot. Tasks are allocated based on the bid responses of the Robots. The Robot with the lowest chance of failure bid is assigned the task. The main difference between the centralised architecture and the decentralised architecture with regard to task allocation is that, whilst the centralised architecture considers all the tasks in a given request, the centralised architecture does not. We have chosen to model task allocation and execution, as well as resource management and communication in this paper to reduce the complexity of the system. The following paragraphs detail the implementation of the decentralised architecture in comparison to the centralised architecture.

Operator: the Operator is identical to the Operator of the centralised architecture.

Requests Manager: the Requests Manager is identical to the Requests Manager of the centralised architecture.

**FIGURE 7:**
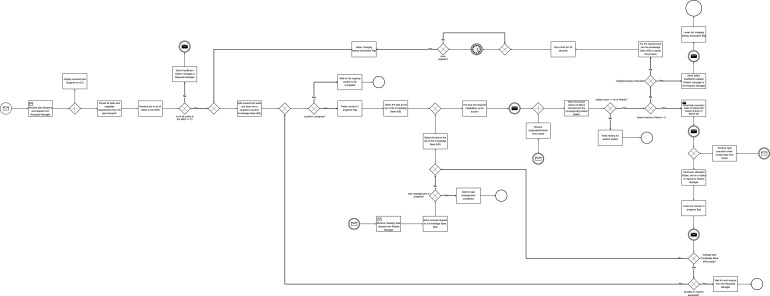
Decentralized architecture: Planner activity diagram.

Planner: responsible for assigning tasks to Robots. This is done by auctioning each task in the request. Figure 7 shows the activity diagram of the Planner for the decentralised architecture. The Planner splits the request into separate tasks and auctions each task and its corresponding execution requirements. The development of the verified plan fails if the MRT has fewer than two Robots. In order to introduce redundancy into the system, we define that there must be more than one Robot available for request execution. Each Robot will either bid for the task or refuse to bid. All the Robots that bid for the task will bid with chance of failure values for the task. The task is therefore allocated to the Robot with the lowest chance of failure. Information about the Robot, allocated task, and the number of tasks in the request are sent to the Robots Manager after each bidding process. The bidding process is repeated until all the tasks in the request are allocated.

The Planner also listens for ‘task reallocation’ messages. A task may be reassigned if it fails or if the allocated Robot deregisters due to low battery. To reassign the task, the planner puts the task up for bidding and the process is repeated. After all tasks have been reassigned, the Planner waits for the next Plan Blueprint to process.

**FIGURE 8:**
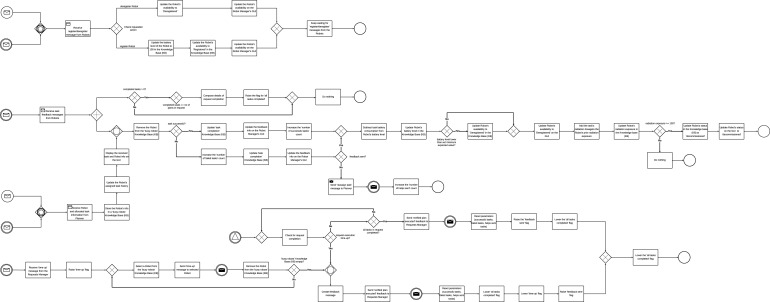
Decentralized architecture: Robots Manager activity diagram.

Robots Manager: The Robots Manager in the decentralised architecture is only responsible for registering and deregistering robots, receiving task feedback from Robots, sending the reassign task message to Planner and sending the request execution feedback to the Requests Manager. The activity diagram in Figure 8 shows the information flow and processes involved.

The Robots Manager receives information about the allocated task and corresponding Robots from the Planner and stores the details noting the Robots as busy. After completing a task, each Robot sends task feedback to the Robots Manager which updates the information received on its GUI and in the KB. If the task execution is successful, the number of successful tasks count is increased. However, if the task fails the number of failed tasks is increased. If the request execution time has not elapsed, the failed task is sent back to the Planner for reassignment. After each task execution, battery levels and radiation exposure are updated on the GUI and KB. If the battery level falls below the minimum set value, the Robots Manager deregisters the robots from the MRT. If the radiation exposure get higher than the expected value, the robot is decommissioned and can no longer be assigned a task.

Simultaneously, the Robots Manager also receives “time-up” messages from the Requests Manager and raises the “time-up” flag. Using the information in the “busy” Knowledge Base (KB), the Robots Manager sends ‘time-up’ messages to all the currently active robot to stop task execution and the task execution fails. If the “time-up” flag is raised during request execution, all active task executions are stopped and reported as failed to the Requests Manager. However, if all tasks are executed before the request execution elapses, a ‘successful’ message is sent to the Request Manager.

If the Robots Manager receives register/deregister messages from any robot. It processes the registering and deregistering processes by updating the KB and GUIs.

**FIGURE 9:**
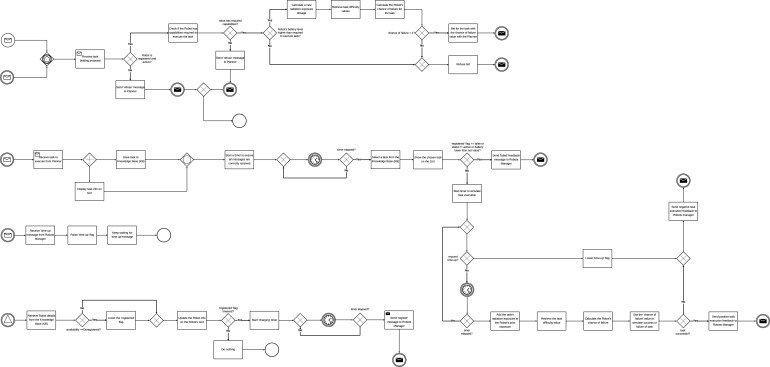
Decentralized architecture: Robot activity diagram.

Robot: Unlike in the centralised architecture, each Robot is involved with the allocation of the task it executes (Figure 9). After receiving the proposal from the Planner, the Robot checks to ensure that it has enough battery power to complete the task. The Robot also checks to confirm that it has the required capabilities to execute the task before calculating its chance of failing to execute the task successfully. The Robot then bids for the task if the calculated chance of failure is less than 1. The proposal is refused if the robot is unregistered, if it does not have the required capabilities, or if the calculated chance of failure is more than 1. If the Robot wins the bid, the Planner sends the task to the Robot. The robot subsequently executes all the tasks it has been assigned. Task execution is simulated using a timer. The duration of the timer varies with the task. As the timer counts, the Robot checks if a ‘time up’ flag has been raised. If the flag is raised before the completion of task execution, the Robot aborts the task and sends a ‘task fail’ feedback to the Robots Manager. When the timer elapses, the Robot calculates the chance of failure and simulates task execution result. To simulate the result, we use a random number generator to generate double values between 0 and 1. The values were generated randomly as random number generators are important in the simulation of real-world processes. Also, each value generated has an equal chance of being generated. If the number randomly generated is greater than the chance of failure, the task succeeds, else the task fails and the feedback is sent to the Robots Manager.

Simultaneously, the robot also frequently updates its details on its GUI by retrieving those details from the KB. If the Robot’s availability reads “Deregistered”, the “registered” flag is lowered and a timer is started to simulate the charging process. When the timer elapses, a “register” message is sent to the Robots Manager to add the Robot to the MRT.

Table 1 shows the comparisons of the different components for centralised and decentralised architectures. in Table 4, we explain the simulation parameters and the rationale behind each of them.

**TABLE 1:** Comparing the system components for Centralised and decentralised architectures.

Component	Centralised architecture	Decentralised architecture
Overall	Single control agent with knowledge of all the robots and their states	This does not operate as a single control agent because the planner does not have knowledge of all available robots and their states
Operator	Sends requests to Requests Manager and receives request execution feedback	Same as in centralised architecture
Requests Manager	Identifies relevant plan blueprint for request execution and sends to Planner with the request	Same as in centralised architecture
Planner	Develops a verified plan with the plans blueprint received from the Requests manager. To develop a verified plan, the Planner retrieves information on all available robots, including their capabilities. The information is used to calculate each robot’s chance of failure when executing the task. The Robot with the lowest chance of failure is assigned the task. Allocated tasks and their corresponding assigned robots make up the verified plan. The Planner also receives task reassignment messages from the Robots Manager if a task fails within the request execution time	Responsible for assigning tasks to Robots by auctioning tasks and their corresponding execution requirements from the plan blueprint. Robots bid for each task with their self calculated chance of failure values. The robot with the lowest value is assigned the task. Information about the Robot, allocated task, and the number of tasks in the request are sent to the Robots Manager. For tasks reassignments, the Planner puts each task up for auction
Robots Manager	The Robots Manager manages the KB of Robots information. Receives the verified plan and assigns tasks to their corresponding robots. The Robots Manager also manages the registering and deregistering of robots from the MRT.	Unlike the centralised architecture, the Robots Manager is not responsible for allocating tasks to the robot but is only responsible for registering and deregistering Robots, receiving task feedback from Robots, sending the reassign task message to the Planner, and sending the request execution feedback to the Requests Manager
Robot	The Robot executes tasks that it has been allocated by the planner based on its capabilities	The robot partakes in the allocation of tasks it is assigned to execute by biding for tasks with its chance of failure for each auctioned task

### 3.4 Use cases

We explored three different use cases for the two architectures. These use cases were designed to model most real-world scenarios, as different organizations and application areas have specific human-in-the-loop requirements. Additionally, the presence or absence of human-in-the-loop involvement can affect task execution difficulty, thereby increasing or decreasing the chances of request execution success.

Use case “No Human Interference”: the two architectures described earlier are modelled without any human interference to complete specific requests. For each task, we have defined task attributes which include task completion time (in seconds), task radiation dosage, and task difficulty. Task completion time was simulated using the sleep function for the duration allocated to completing the task. Since we are exploring the use of MRTs in nuclear environments, we have also allocated radiation dosage to each task based on the nature of the task (e.g., 5, 10). For example, we assume that a mapping task will expose the robot to a lower radiation dosage than a task which requires the robot to move barrels from one place to the other. Lower and higher numbers are assigned to tasks with lower and higher radiation exposures respectively. The maximum cumulative radiation dosage a robot should be exposed to in this simulation is 100, after which the robot is decommissioned and can no longer be assigned any task. The higher the task difficulty, the higher the numbers assigned (e.g. 20, 30, 40, … , 80). We assume that a robot may fail to complete a task due to operational failures caused by mechanical issues, software glitches, environmental conditions, human interference, or control system malfunctions, as well as challenges associated with the task itself. The success of each task was therefore calculated as a function of cumulative radiation exposure, which may contribute to the causes of failure highlighted and task difficulty which may both be reduced with the introduction of a human collaborator.

As shown in Equation 1, a new cumulative radiation exposure dosage $\left(Dosage_{\mathrm{nce}}\right)$ of a robot carrying out a task is calculated by adding the radiation exposure dosage of the task $\left(Dosage_{t}\right)$ to the robot’s previous cumulative exposure dosages $\left(Dosage_{\mathrm{pce}}\right)$ .

$$Dosage_{\mathrm{nce}}=Dosage_{\mathrm{pce}}+Dosage_{t}$$ (1)

Setting the maximum possible values of the cumulative exposure dosage and task difficulty to 100 respectively, the sum of both parameters should yield a maximum value of 200. We have calculated the chance of failure for each task following Equation 2.

$$Failure_{\mathrm{chance}}=\frac{Dosage_{\mathrm{nce}}+Difficulty_{\mathrm{task}}}{200}$$ (2)

This means that the maximum value for the chance of failure will always be 1. Therefore the chance of successfully carrying out each change will be inputted into the simulation as

$$Success_{\mathrm{chance}}=1-Failure_{\mathrm{chance}}$$ (3)

Use case “Human Collaboration”: in this use case, the operator collaborates with the MRT by taking over a robot after the tasks have been assigned to complete the task assigned to the robot (Freedy et al., 2007; Desai et al., 2013; Xu and Dudek, 2015). Taking over after the tasks have been assigned ensures that the operator is not involved with task allocation but only collaborates with the team of robots to execute a request. The difference with the chance of failure in tasks executed in this use case however is that task difficulty is lowered to a third. We assume that having the operator tele-operate a robot in executing a task reduces the task difficulty, and in this case, making it a third of the difficulty. Therefore the chance of failure is calculated as shown in Equation 4.

$$Failure_{\mathrm{chance}}=\frac{Dosage_{\mathrm{nce}}+\left(Difficulty_{\mathrm{task}}\right)/3}{200}$$ (4)

The chance of success remains as defined in Equation 3.

The Operator in this use case only receives the information about the paired robots and their allocated tasks from the Robots Manager and sends a reply with a randomly selected robot and its allocated task. The Operator also sends a ‘take over’ message to the selected robot. The changes described for the human collaboration use case also applies to the decentralised architecture for the Operator, Robots Manager, and Robot.

Use case ’Human Intervention’: for the human intervention use case, an operator takes over a robot after tasks have been allocated for an entirely different purpose, causing the task to fail as a result. The task is therefore reassigned to another robot or put out for auction (dependent on architecture). We have also assumed that all tasks execution must be successful for the request execution to be successful.

## 4 Evaluation

In order to evaluate the impact of human collaboration, human intervention, and number of robot on MRTs with centralised and decentralised architectures, we set up 4 simulation runs (i.e. each scenario with different numbers of robots in the MRT). Each scenario (centralised architecture with no human involvement (C), centralised architecture with human collaboration (C_HC), centralised architecture with human intervention (C_HI), decentralised architecture with no human involvement (D), decentralised architecture with human collaboration (D_HC), and decentralised architecture with human intervention (D_HI)) is a combination of MRT architecture and human involvement. Each simulation run was repeated 40 times for all scenarios which are combinations of system architectures and use cases (2 architectures x 3 use cases), as well as different numbers of robots in the MRT (4, 6, 8, and 10 robots). In this Section we first show how robot capabilities, tasks, request to MRT, plan blueprints, and robots were set up for the evaluation (Section 4.1). We then present the dependent measures used to measure the simulation runs (Section 4.2), and the evaluation results (Section 4.3).

### 4.1 Evaluation setup

We begin by defining capabilities for the robots based on the tasks to be executed. We also define robot tasks that are commonly executed in nuclear facilities. Finally, we link each task with the capabilities required for each robot to be able to execute it. Table 2 shows capabilities, tasks, and how they are linked.

**TABLE 2:** Evaluation setup. Robot capabilities, tasks, and link between both.

Capability		Task		Task-capability link
$C_{1}$	proximity sensor	$T_{1}$	map an area	$T_{1}$ : $\langle C_{1}$ , $C_{4}$ , $C_{6}\rangle$
$C_{2}$	radiation sensor	$T_{2}$	identify locations of high radiation	$T_{2}$ : $\langle C_{1}$ , $C_{2}$ , $C_{4}$ , $C_{6}\rangle$
$C_{3}$	gripper	$T_{3}$	move barrels	$T_{3}$ : $\langle C_{1}$ , $C_{2}$ , $C_{3}$ , $C_{4}$ , $C_{5}$ , $C_{7}\rangle$
$C_{4}$	camera	$T_{4}$	connect device to power source	$T_{4}$ : $\langle C_{1}$ , $C_{3}$ , $C_{4}$ , $C_{5}$ , $C_{7}\rangle$
$C_{5}$	gyroscope			
$C_{6}$	lidar			
$C_{7}$	bump sensor			

Additionally, we define a number of requests issued to the MRT. In each simulation attempt, nine requests are executed. Each request comprises of several combinations of tasks to be executed. We also define plan blueprints that describe how each request may be executed by listing the tasks the MRT needs to execute. Finally, we assigned different capabilities to a set of robots that represent a possible MRT. The team composition was developed such that there will always be enough robots to execute all tasks in a given request unless a robot leaves the MRT to charge its batteries, or has been decommissioned having being exposed to the maximum radiation dose it can withstand. Table 3 shows all requests, plan blueprints, and robots with their capabilities.

**TABLE 3:** Requests, plan blue prints, and MRT composition.

Request	Plan blueprint	Robot (capabilities)
$RQ_{1}$ : $\left\langle T_{1},T_{2}\right\rangle$	$PB_{1}$ : $\left\langle T_{1},T_{2}\right\rangle$	$R_{1}$ : $\left\langle C_{1},C_{4},C_{6}\right\rangle$
$RQ_{2}$ : $\left\langle T_{1},T_{2},T_{4}\right\rangle$	$PB_{2}$ : $\left\langle T_{1},T_{2},T_{4}\right\rangle$	$R_{2}$ : $\left\langle C_{1},C_{2},C_{4},C_{6}\right\rangle$
$RQ_{3}$ : $\left\langle T_{1},T_{3},T_{4}\right\rangle$	$PB_{3}$ : $\left\langle T_{2},T_{3}\right\rangle$	$R_{3}$ : $\left\langle C_{1},C_{2},C_{3},C_{4},C_{5},C_{7}\right\rangle$
$RQ_{4}$ : $\left\langle T_{1},T_{4}\right\rangle$	$PB_{4}$ : $\left\langle T_{2},T_{3},T_{4}\right\rangle$	$R_{4}$ : $\left\langle C_{1},C_{3},C_{4},C_{5},C_{7}\right\rangle$
$RQ_{5}$ : $\left\langle T_{1},T_{3}\right\rangle$	$PB_{5}$ : $\left\langle T_{1},T_{3}\right\rangle$	$R_{5}$ : $\left\langle C_{1},C_{2},C_{3},C_{4},C_{5},C_{6},C_{7}\right\rangle$
$RQ_{6}$ : $\left\langle T_{1},T_{2},T_{3}\right\rangle$	$PB_{6}$ : $\left\langle T_{1},T_{2}\right\rangle$	$R_{6}$ : $\left\langle C_{1},C_{4},C_{5},C_{6}\right\rangle$
$RQ_{7}$ : $\left\langle T_{2},T_{3}\right\rangle$		$R_{7}$ : $\left\langle C_{1},C_{2},C_{3},C_{4},C_{6}\right\rangle$
$RQ_{8}$ : $\left\langle T_{2},T_{3},T_{4}\right\rangle$		$R_{8}$ : $\left\langle C_{1},C_{3},C_{4},C_{5},C_{7}\right\rangle$
$RQ_{9}$ : $\left\langle T_{2},T_{4}\right\rangle$		$R_{9}$ : $\left\langle C_{1},C_{2},C_{3},C_{4},C_{5},C_{6},C_{7}\right\rangle$
		$R_{10}$ : $\left\langle C_{1},C_{3},C_{4},C_{5},C_{6},C_{7}\right\rangle$

In order to make the system behaviour dynamic, we also introduced variations into the system. The variations ensure that the results of each simulation varies based on the characteristics of the agents in the system. The variations include:

Plan blueprint: the plan blueprint provides information on the capabilities a robot must possess to execute tasks in a request, and could be available or unavailable. If no plan blueprint is found for a specific request, the request execution fails. We implemented the simulation to demonstrate the two possible scenarios.

Varying numbers of registered/unregistered robots in the MRT: the simulation was designed to allow robots to dynamically register and deregister themselves from the MRT. A robot deregisters itself from the MRT if its battery level drops below a certain level and registers when the battery is charged. For every task executed, a robot’s battery level drops. The amount of drop depends on the task executed.

Varying numbers of active robots: the dynamic behaviours of the robots in the MRT imply that we could have varying numbers of active robots as requests are executed. This is because robots can either temporarily deregister themselves when their battery levels are low or get decommissioned if their radiation exposure exceeds the allowed level. This creates the possibilities of having varying numbers of active robots in the MRT as requests are executed. The number of robots in the MRT are the same at the beginning of each simulation instance.

Capabilities of each robot: in real-world scenarios, different robots may have different capabilities to make them suitable for varying tasks. We have modelled the robots in our study to have varying capabilities.

Operator involvement: three scenarios of operator involvements were explored. The first scenario involves the robots executing tasks with no human operator involvement. In the second scenario, the human collaborates with the robots to execute tasks after the tasks have been allocated. In the third scenario, the human intervenes by taking over a robot to execute a separate task than was allocated to the robot.

Different architectures (task allocation differences): centralised and decentralised architectures were explored. In the centralised architecture, the system has access to all the information about all robots in the MRT and allocates tasks based on the information. In the decentralised architecture, tasks are auctioned and each robot bids for the task with its calculated chance of failure.

Varying request compositions: requests are made up of two or more different tasks. The task composition in each request also vary, creating different request execution outcomes.

### 4.2 Dependent measures

In order to compare the different scenarios, we identified dependent measures with which comparisons can be made to examine the performance of scenarios. Table 4 gives detailed information about the measures used in our simulations.

**TABLE 4:** Dependent measures for MRT Simulation.

Component	Centralised architecture
Request outcome	The execution of a request can either be a “success” or “failure”. A request may fail due to various factors, some of which we have examined in this paper. The first factor is the unavailability of plan blueprints to execute the tasks in the request. A second factor is the unavailability or low number of sufficient and capable robots in the MRT to handle the tasks. Robots in the MRT may be unavailable having been de-registered for charging or decommissioned due to excessive exposure to radiation. The chance of robots getting de-registered or decommissioned may increase if there is a low number of robots in the MRT, or due to continuous task failure and the need for task reassignment. As requests have to be executed within a specific time, a request execution may also fail if all the tasks in the request are not successfully executed within the given time frame. Finally, different levels of human involvement may also affect request execution outcomes since there is no guarantee that human involvements will always lead to successful request execution. Advantages of using request outcomes as a performance metric include scenario analysis, system improvement and resource management
Task outcome	Each request to be executed contains tasks, and each task execution can result in either failure or success. Since we are simulating the behaviours of each agent, no actual tasks are performed; instead, a loop is used to count down the task execution time. After each countdown, a new chance of failure is calculated, and a random number generator is used to produce values between 0 and 1. These values are appropriate because the chance of failure cannot exceed 1 or be less than 0. If the generated random number is greater than the calculated chance of failure, the task succeeds. This method ensures that a higher chance of failure makes it more difficult for the task to succeed. The number of failed and successful tasks within each request execution is recorded. If not all tasks in a request are successfully executed before the specified request execution time elapses, the request execution fails. Additionally, within that time frame, a task may fail, be reassigned to another robot, and then be successfully executed after reassignment. This is why the number of successful and failed task executions is recorded for all scenarios examined
Request completion time	This metric measures the time taken to complete the execution of a request. A request fails if the timer runs out before all tasks in the request are completed or if there are no plans blueprint to execute the request. If a request fails due to the unavailability of plans blueprint, RCT is recorded as zero. However, if a request fails due to the timer running out, the allocated request execution time is recorded. Since the task allocation process varies across different architectures, and varying levels of human involvement can impact task execution outcomes, it is crucial to measure the duration required to execute requests

### 4.3 Results

We conducted the simulation 40 times for each scenario, which included the independent variables: MRT architecture (centralised and decentralised), number of robots (4, 6, 8, 10) in the MRT, and level of human involvement (no human involvement, human collaboration, and human intervention). Table 5 shows the results across scenarios for the request outcome (no. of successful requests), task outcome (no. of successful tasks and failed tasks), and request completion time.

**TABLE 5:** Evaluation results for all scenarios.

Scenario	Robots	No. successful requests		No. successful tasks		No. failed tasks		Request completion time	
		mean	SD	mean	SD	mean	SD	mean	SD
C: centralised architecture	4	5.85	0.700	14.73	0.877	9.50	3.566	273.93	36.544
	6	6.32	0.764	15.23	0.947	7.40	3.622	251.28	39.491
	8	6.45	0.597	15.40	0.709	6.20	2.653	244.38	28.556
	10	6.45	0.677	15.32	0.971	6.33	2.965	236.38	33.695
C_HC: centralised architecture human collaboration	4	6.37	0.705	15.15	1.210	5.25	3.028	246.68	32.904
	6	6.75	0.439	15.75	0.439	4.18	2.135	222.10	23.872
	8	6.65	0.483	15.53	0.784	4.55	2.353	229.55	23.834
	10	6.88	0.404	15.83	0.675	3.55	2.207	211.73	25.211
C_HI: centralised architecture human intervention	4	3.85	1.122	11.22	1.874	17.90	2.790	315.95	42.888
	6	5.57	0.874	14.33	1.207	18.55	4.266	301.25	36.060
	8	6.35	0.700	15.17	1.010	15.00	3.382	271.65	29.955
	10	6.20	0.992	14.90	1.297	14.18	3.551	263.35	33.388
D: decentralised architecture	4	5.85	0.700	14.65	1.122	8.35	3.527	251.60	31.740
	6	6.22	0.660	14.98	1.209	6.55	3.071	243.60	29.828
	8	6.50	0.679	15.45	0.846	5.55	2.669	239.55	27.852
	10	6.32	0.656	15.18	0.984	6.45	3.021	243.53	32.429
D_HC: decentralised architecture human collaboration	4	6.45	0.714	15.43	0.747	6.00	3.623	245.55	34.127
	6	6.68	0.526	15.67	0.526	3.90	3.463	216.52	30.277
	8	6.68	0.526	15.58	0.781	3.30	2.198	226.48	25.919
	10	6.60	0.545	15.57	0.594	3.45	2.396	219.13	23.724
D_HI: decentralised architecture human intervention	4	4.63	1.295	10.03	2.537	14.53	2.582	343.17	47.132
	6	5.60	1.033	14.35	1.388	17.60	3.380	290.20	35.691
	8	5.95	0.749	14.58	1.217	16.78	2.815	282.77	29.052
	10	6.10	0.810	14.98	0.974	14.68	3.562	259.63	30.628

#### 4.3.1 Request execution outcome

**FIGURE 10:**
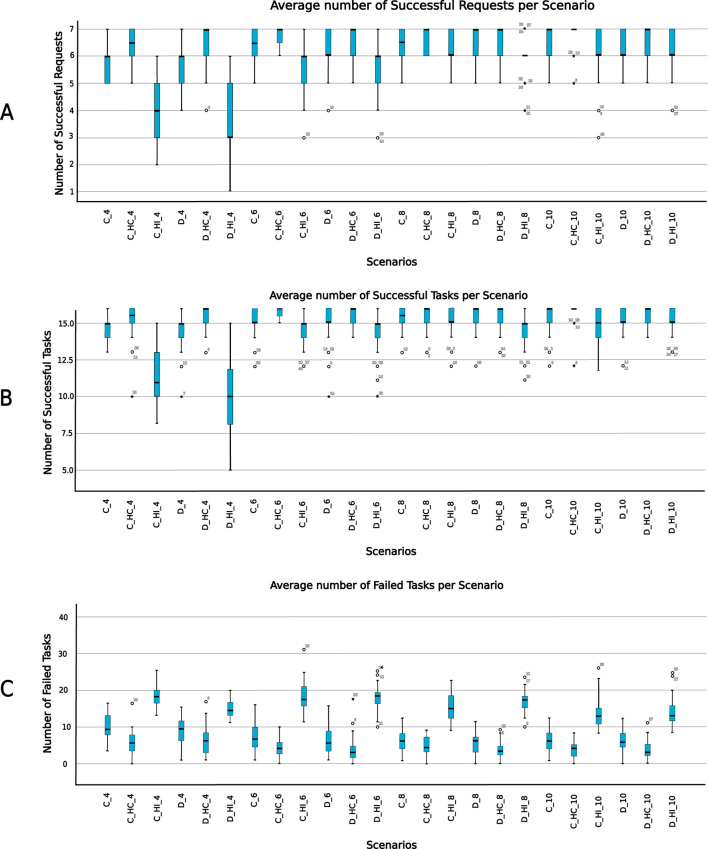
Simulation outcome. **(A)** Average number of successful requests. **(B)** Average number of successful tasks. **(C)** Average number of failed tasks. Labels follow the convention: [architecture type (C / D)] _ [use case (NO/HI/HC)] _ [number of robots (4/6/8/10)].

By design, each scenario can have a maximum of 7 successful requests. From the 9 requests the Operator sends, 2 request executions fail because there are no plan blueprints for their execution. The descriptive statistics of the average number of successful requests for each scenario are shown in Table 5. Figure 10A shows the average number of successful requests for all scenarios. Since each request execution involves the execution of tasks within the request, it is important to likewise examine task execution outcomes.

#### 4.3.2 Task execution outcome

The overall number of tasks in each simulation run was consistent across all scenarios, but the number of successfully executed tasks varied between scenarios (Figure 10B). Given sufficient time, and provided there are capable robots, all tasks in a request will eventually be successfully completed provided there are plan blueprints for their execution. However, since real-world applications do not allow for indefinite time or an unlimited number of capable robots to execute tasks, the number of successful task executions varied across scenarios. Table 5 shows the descriptive statistics of the average number of successful tasks for all scenarios. It is therefore important to examine the number of task execution failures in each scenario, as this can serve as a key metric for differentiating between the scenarios. Table 5 also shows descriptive statistics of the average number of failed tasks. Figure 10C shows the boxplots of failed tasks for all scenarios.

Shapiro-Wilk test of normality was conducted on the average number of failed task executions under both architecture conditions (centralised (W(480)=.979,p<.001) and decentralised (W(480)=.946,p<.001) , number of robots in the MRT condition (4 (W(240)=.972,p<.001) , 6 (W(240)=.923,p<.001) , 8 (W(240)=.920,p<.001) , 10 (W(240)=.944,p<.001) ), and levels of human involvement (none (W(320)=.978,p<.001) , human collaboration (W(320)=.918,p<.001) , and human intervention (W(320)=.976,p<.001) )). The results indicated a significant deviation from normality. A Mann-Whitney U test was conducted to investigate the effect of the MRT architecture on the number of failed tasks. The difference in the number of failed tasks between the two architectures was not statistically significant U=109,961.000,Z=−1.22,p=.222 . We conducted a Kruskal–Wallis H test to examine the differences in the number of failed tasks for different numbers of robots (4,6,8,10) in the MRT. The results show that the distribution of failed tasks was significant between the groups H(3)=20.86,p<.001 . For different levels of human involvement, the Kruskal–Wallis H test showed a statistically significant difference in failed tasks between groups H(2)=646.46,p<.001 .

**FIGURE 11:**
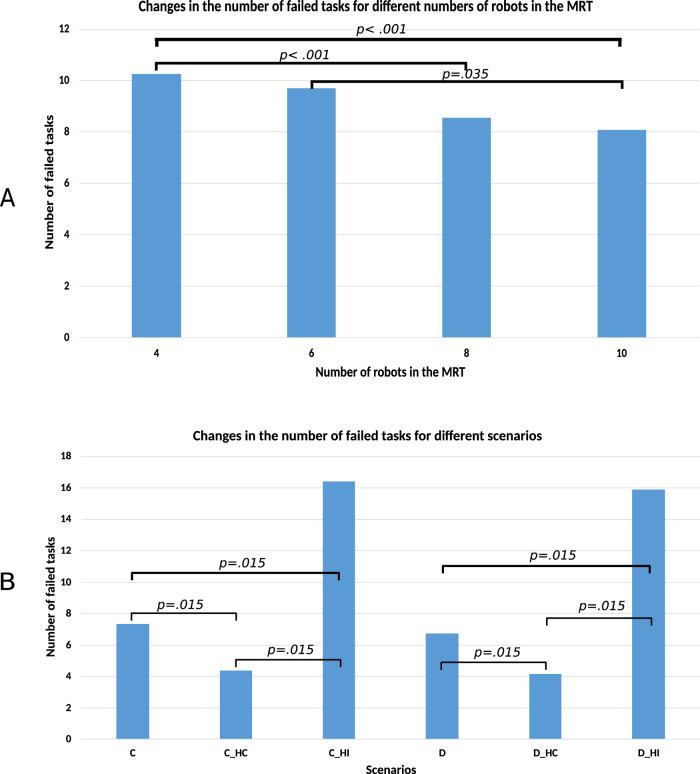
Mean number of failed tasks. **(A)** between different numbers of robots in the MRT **(B)** between different scenarios.

We conducted Mann-Whitney test to compare the number of failed tasks between different number of robots in the MRT. Results as follows: 4 and 6 robots in the MRT ( U=26,108.00,Z=−1.77,p=.076 , indicating that there is no significant difference in failed tasks), 4 and 8 robots in the MRT ( U=23,438.00,Z=−3.53,p<.001 , indicating that there is a statistically significant difference in the number of failed tasks), 4 and 10 robots in the MRT ( U=22,188.00,Z=−4.36,p<.001 , indicating that there is a statistically significant difference in the number of failed tasks), 6 and 8 robots in the MRT ( U=26.560.50,Z=−1.48,p=.140 , indicating that there is no significant difference in the number of failed tasks), 6 and 10 robots in the MRT ( U=25,593.50,Z=−2.11,p=.035 , indicating that there was a statistically significant difference in the number of failed tasks), 8 and 10 robots in the MRT ( U=27,756.50,Z=−0.69,p=.491 , indicating that there no significant difference in the number of failed tasks). Figure 11 shows the plot of the mean failure number for varying numbers of robots and scenarios. Only significant differences are shown. Increasing the number of robots is advantageous, but only up to a certain point. Post hoc pairwise comparisons of the number of failed tasks for different levels of human involvement (no, HC, and HI), using Bonferroni adjustment, showed a significant difference between all pairs of groups. Trials without human involvement had more failed tasks than the trials with human collaboration (Mean difference = 2.77, SE = 0.26, p<.001 ), but fewer than trials with human intervention (mean difference = −9.11, SE = 0.26, p<.001 ). Furthermore, the human collaboration group had fewer failed tasks than the human intervention group (mean difference = −11.88, SE = 0.26, p<.001 )

For the combination of system architecture and levels of human involvement (C, C_HC, C_HI, D, D_HC, and D_HI), normality tests using the Shapiro-Wilk test indicated that the assumption of normality was violated for all six groups. A Kruskal–Wallis test revealed a statistically significant difference in failed tasks among the six architecture - human involvement role groups, $\chi^{2}\left(5\right)=648.21,p<.001$ . Mann-Whitney U tests were carried out on failed tasks between the groups. Figure 11B shows the significant comparisons.

#### 4.3.3 Request completion time

**FIGURE 12:**
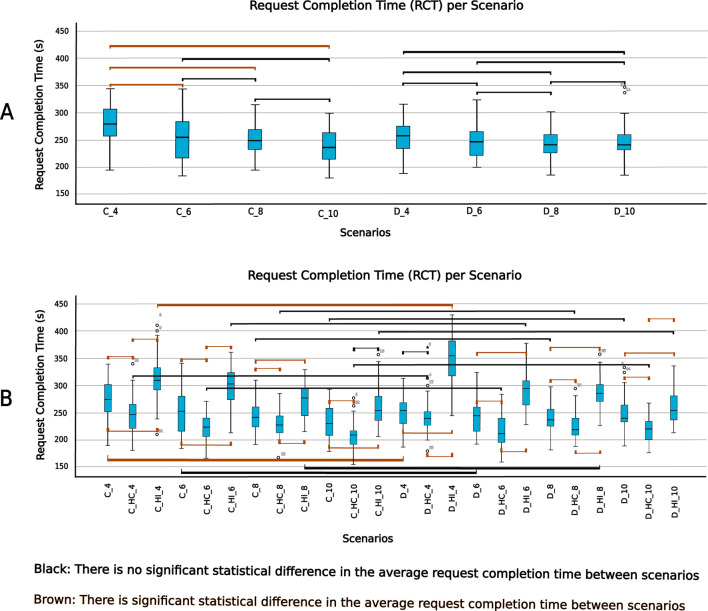
Paired Samples t-test **(A)** scenarios without human involvement for request completion time **(B)** scenarios with human involvement for request completion time.

We define the Request Completion Time (RCT) as the time taken to complete each request. RCT depends on the number of task execution failures, which could be another significant factor distinguishing each scenario. An ideal scenario would involve all tasks being successfully executed on first attempt, in which case RCT should be identical across all scenarios. However, this is not the case since scenarios affect task execution outcomes. We set the maximum allowable value for RCT at 60 s; requests exceeding this time limit are considered failed. Figure 12 shows the boxplots of the request completion time for all scenarios.

Test of normality shows that the average request completion time for the different scenarios explored followed a normal distribution. Mauchly’s test, $\chi^{2}\left(5\right)=6.212,\;p=0.286$ did not indicate any violation of sphericity for the number of robots in the MRT. However, Mauchly’s test, $\chi^{2}\left(14\right)=30.095,\;p=0.008$ indicated violation of sphericity for the scenarios. The interaction between the number of robots and scenario also violated the assumption of sphericity $\chi^{2}\left(119\right)=163.662,\;p=0.006$ .

A linear mixed-effects model was used to examine the effects of architecture, human involvement, the number of robots in the MRT and their interactions on requests completion time. The analysis revealed a significant main effect of different levels of human involvement (F(2,936)=322.778,p<.001) , and the number of robots in the MRT (F(3,936)=68.037,p<.001) . However, there was no significant effect of architecture on request completion time (F(1,936)=.067,p=.796) .

The interaction between architecture and levels of human involvement (scenarios) (F(2,936)=3.131,p=.044) , as well as between human involvement and number of robots in the MRT (F(6,936)=9.830,p<.001) was significant. However, the architecture by number of robots in the MRT interaction was not significant (F(3,936)=1.529,p=.205) . In addition, the three-way interaction between system architecture, levels of human involvement, and the number of robots in the MRT was significant (F(6,936)=3.676,p=.001) . This indicates that the combined effect of human involvement and number of robots in the MRT on the completion time of requests depended on the architecture.

Pairwise comparisons of estimated marginal means (with Bonferroni correction) showed significant difference in requests completion time across all levels of human involvement. Simulation trials with human intervention condition (HI) had significantly longer completion times than trials in human collaboration condition (HC) (mean difference = 63.78, SE = 2.56, p<.001 , 95% CI [57.64, 69.92]). The condition without any form of human involvement had significantly shorter completion time than the HI condition (mean difference = 42.97, SE = 2.56, p<.001 , 95% CI [36.83, 49.11]). The human collaboration condition (HC) resulted in significantly faster request completion time than both HI (mean difference = −63.781, SE = 2.56, p<.001 , 95% CI [-69.92,-57.64]) and the scenario without human involvement (mean difference = −20.812, SE = 2.56, p<.001 , 95% CI [-26.953, −14.672])

**FIGURE 13:**
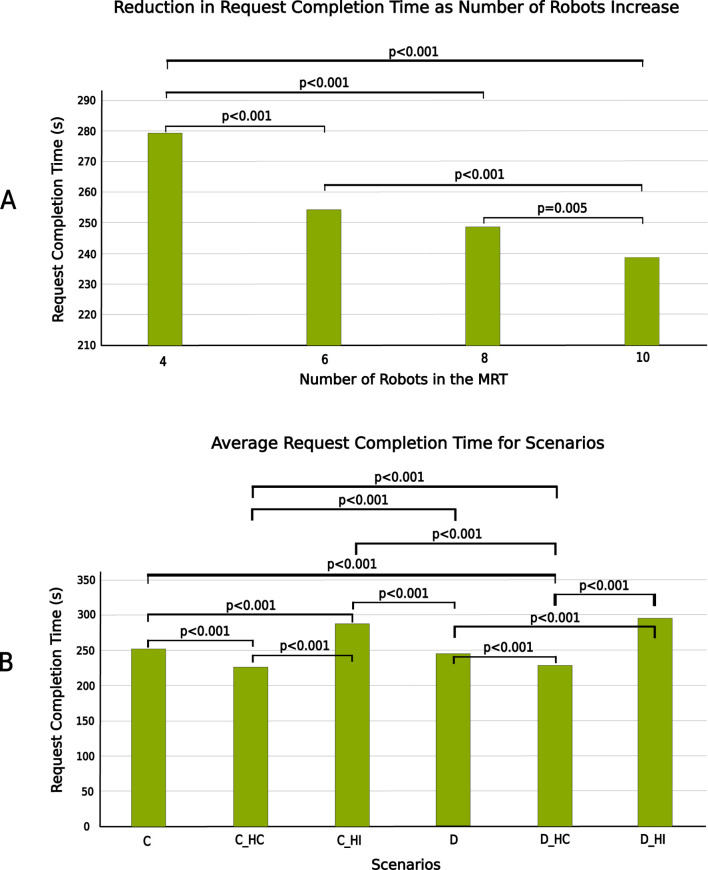
Mean request completion time **(A)** between different numbers of robots in the MRT **(B)** between different scenarios.

A *post hoc* pairwise comparison using the Bonferroni correction was also carried out between the different instances of number of robots and scenarios. Figures 13A,B show the plot of average request completion time for varying numbers of robots and scenarios respectively. They also show the significance of pairwise comparisons. Only the significant differences are shown.

We further compared all the scenarios using a paired samples t-test for the different numbers of robots in the MRT and their corresponding scenarios. Figures 12A,B show the paired samples t-test carried out to compare all the scenarios for the different categories of robots in the MRT.

## 5 Discussion

Results show that the number of robots in the Multi-Robot Team (MRT) was a significant factor influencing task execution success or failure, and in return request execution success. By increasing the number of robots in the MRT, we were able to consistently reduce task failures. This may be due to the cumulative effects of radiation exposure on robots, which impacts their success in task execution, as radiation dosage is factored into calculating each robot’s failure probability. As the number of robots increases, especially with the addition of those with similar capabilities, tasks are assigned to robots with a lower chance of failure, thereby reducing the number of failed tasks. With the introduction of human collaboration and human intervention, results showed that they had different effects on the number of failed tasks. While human collaboration reduced the number of failed tasks, human intervention significantly increased the number of failed tasks. The significant difference in the number of failed tasks between scenarios with human collaboration and human intervention is as a result of the human operator taking over robots to execute tasks different from their originally assigned tasks, leading to the failure of the assigned tasks.

Similarly, number of robots in the MRT significantly affected the request completion time (RCT). Generally, increasing the number of robots in the MRT reduced the RCT. As the number of robots in the MRT increases, task execution failures decrease, which in turn reduces the request completion time, as tasks will not need to be reallocated to other robots for completion. Specifically, for centralised architectures, increasing the number of robots significantly reduced the RCT, whereas this effect was not observed in decentralized architectures. Human collaboration reduced the request completion time (RCT), while human intervention increased the RCT, regardless of the number of robots in the MRT. Keeping the number of robots in the MRT constant, there was a significant difference in request completion times between centralised and decentralised architectures when the number of robots was low. However, as the number of robots in the MRT increased, the request completion times for centralised and decentralised architectures became similar. The reason for the differences in RCT between the two architectures with lower number of robots in the MRT may be because centralised architecture has more access to system data and may be able to allocate tasks better, especially when more than one task has to be allocated to a robot. This may be investigated further in future studies.

Given sufficient time and an unlimited number of capable robots in the MRT, all requests will eventually be successfully executed. However, in real-world scenarios, MRTs do not have indefinite request execution time and unlimited numbers of capable robots. As such, decisions must be made regarding system architecture, number of robots in the MRT, and different levels of human involvement. As the number of robots in the MRT increases, the number of successful request executions also increases. Request executions fail if all tasks within a request are not successfully executed within the allocated time.

Since task allocations are made by calculating the robot’s chance of failure, which depends on factors such as radiation exposure, task difficulty, and the level of human involvement, having a higher number of robots in the MRT ensures that only robots with higher chances of success are assigned tasks. Conversely, if the number of robots in the MRT is low, tasks will be allocated to the available robots that can execute the tasks, leading to significantly increased radiation exposure for the robots. This increased exposure raises the chances of robots getting decommissioned or having to leave the MRT to recharge their batteries. When this happens, if the MRT does not have capable robots to whom the task would be assigned to, the request fails.

Human collaboration reduces the number of failed tasks by reducing the chances of failure. The effect of different levels of human involvement was significant when the number of robots in the MRT was low. This is because when the number of robots in the MRT is low, there are fewer robots to assign task to, which may lead to having to assign more than one task to a robot. When tasks fail, task reassignment will also be more difficult with fewer number of robots in the MRT. Our results showed that having more than 8 robots did not further increase the number of successful task executions in our simulation as that was found to be the limit of redundancies introduced in our simulation by having robots with similar capabilities.

### 5.1 Design recommendations

The simulation results discussed may be used in the formulation of design guidelines for developing human robot interaction - MRTs (HRI-MRT) systems:

1. *If one wants to reduce task failures, increase number of robots, up to a point.* As shown in the results from Section 4.3.2, regardless of the architecture used, a higher number of task failures was observed when fewer robots were involved in the multi-robot team (MRT). However, after a certain threshold, increasing the number of robots in the MRT does not have any significant effect on reducing no of failed tasks.
2. *If you can, avoid human intervention.* As shown in Table 5, scenarios involving human intervention had the highest number of failed tasks and the least number of successful tasks and requests.
3. *Human collaboration decreases task failures and request completion time.* As described in the study, human collaboration reduces the chance of failure in the execution of a task, hence resulting in decreased task failures and request completion time.
4. *Simulations shows problems will occur, so plan for it.* The different scenarios and the number of robots in the MRT will introduce different problems in the system. For instance, human intervention, where a human operator takes control of a robot to perform a task other than its assigned one, can lead to an increase in task failures. This may result in longer request completion times and more failed task executions. Running the simulation can therefore reveal the possible problems that may arise as a result of the different scenarios and no of robots in the MRT.
5. *Redundancies should be implemented to prevent system failures.* We introduced redundancies into the system by increasing the capabilities of each robots and adding robots with similar capabilities into the MRT. Increasing the capabilities of each robot ensures that each robot may be assigned a higher number of tasks, which increases the chance that each request will be executed. Likewise, in increasing the number of robots in the MRT, we introduced robots with similar capabilities to reduce the rate at which robots deregister to recharge or get decommissioned.
6. *Impact of system architecture is higher with lower number of robots in the MRT.* Comparing results for centralised and decentralised architectures in Table 5; Figure 12, the number of failed tasks and the request completion time were significantly higher in centralised architecture than decentralised architecture with 4 robots in the MRT. As the number of robots in the MRT was increased, there was no significant differences in the measured performance parameters between the 2 architectures. Therefore, in order to compare the effect of different architectures, reduce the number of robots in the MRT.

### 5.2 Limitations and future work

Our study was limited by the simulation environment, which does not capture all real-world variables. For example, the simulation environment did not take into account potential hardware challenges that may affect system performance. We suggest that this is taken into account in future research and real-world implementations. In addition, more real-world variables may also be simulated and accounted for. The operator cognitive load may also be simulated to provide better understanding of the interaction between the human and team of robots from the human point of view. Furthermore, to enhance realism, the simulation may incorporate decision variability or the likelihood of errors. To effectively address the research questions posed in this paper, we limited the number of parameters included in the simulation and made assumptions where necessary. Additionally, due to computational constraints, certain behaviors were abstracted and treated as black boxes.

In this paper, we also assumed the effect of factors such as radiation exposure. However, the effects of radiation exposure on real robot hardware are not fully understood and should be further investigated to ascertain how radiation exposure would affect robot performance and the electronic circuitry of the MRT components. This paper also does not explicitly simulate other failure modes such as communication failures, task execution faults, sensor degradation, or timing jitters which are all relevant in real-world multi-robot teams. Although some of these modes were considered when we introduced the chance of failure in the simulation, additional parameters may be introduced to account for these failure modes.

Another limitation of this research is with regard to the system architectures examined. Other architectures of interest may also be simulated in future research for thorough comparisons. In addition, the types of tasks and the level of human involvement were controlled, which may not reflect all operational scenarios.

To improve simulation efficiency, it is important to account for variability in task execution and request completion times, as similar tasks may not be performed identically in real-world settings due to a range of influencing factors. These real-world uncertainties can significantly impact both task execution and task completion times. Variability in task execution and completion times may be introduced into the simulation via stochastic task durations or execution of delay variability.

While we hope that the design and results presented in this paper are applicable to other contexts, it is important to acknowledge the contextual limitations of our approach. Certain simulation design decisions were tailored to the specific requirements of our application domain. Although the overall agent-based design is transferable, adaptations may be necessary to align with the characteristics and constraints of other domains.

## 6 Conclusion and outlook

The need to simultaneously execute tasks in application scenarios where robotic solutions are employed necessitates the use of MRTs. This requirement is also driven by the fact that it is challenging to have individual robots capable of performing all possible tasks, hence the need to employ a team of robots with specialized functionalities. Additionally, robots are designed with features that allow them to operate in specialized terrains with varying levels of autonomy.

Although different levels of autonomy may be employed as robots execute tasks, some use-cases insist on having human involvement in the task execution process. This introduces the possibility of human intervention when there is system failure. One such application that requires a human in the loop is nuclear decommissioning and operations in nuclear power stations, where robot teleoperation is employed to reduce worker exposure to radiation. The safety requirements in such environments necessitate different levels of human involvement, even when teams of robots are employed. Describing how a team of robots would perform with the introduction of a human operator remains a challenging task. Therefore, it is important to investigate how this may affect the task execution performance of MRTs.

We implemented a simulation framework using the Java Agent DEvelopment (JADE) framework to compare the request execution performance of centralised and decentralised architectures, as well as different levels of human involvement (no human involvement, human collaboration, and human intervention). We also compared performance based on different numbers of robots (4, 6, 8, 10) in the MRT. The performance metrics employed include request execution outcomes (success or failure), number of successful tasks, number of failed tasks, and request completion time (RCT).

All relevant components of the human-in-the-loop MRT were modeled as agents with defined functionalities and behaviors. Each agent is able to communicate with all other agents, simulating real-world functionalities. The different agents in our framework include the Human Operator, Requests Manager, Planner, Robots Manager, and Robots.

The design guidelines and findings outlined in Sections 4, 5 can assist researchers and system developers aiming to deploy MRTs in human-in-the-loop scenarios, helping guide their decision-making processes. Additionally, we have shown that simulating different use cases can significantly reduce the cost and time otherwise spent on purchasing robots and implementing MRTs to obtain results.

Future research should focus on exploring varying task complexities and more diverse operational environments. Examining different coordination and task allocation algorithms could also offer valuable insights into optimizing the performance of robot teams.

## Data Availability

The raw data supporting the conclusions of this article will be made available by the authors, without undue reservation.

## Supplementary material

SUPPLEMENTARY FIGURE S1 BPMN Notations used for modelling different architectural scenario.
