# Supplementary figures and images for: Should we get involved? impact of human collaboration and intervention on multi-robot teams
Source: Front Robot AI. 2025 Oct 15;12:1526287. doi: 10.3389/frobt.2025.1526287 (PMC12569544; doi:10.3389/frobt.2025.1526287)

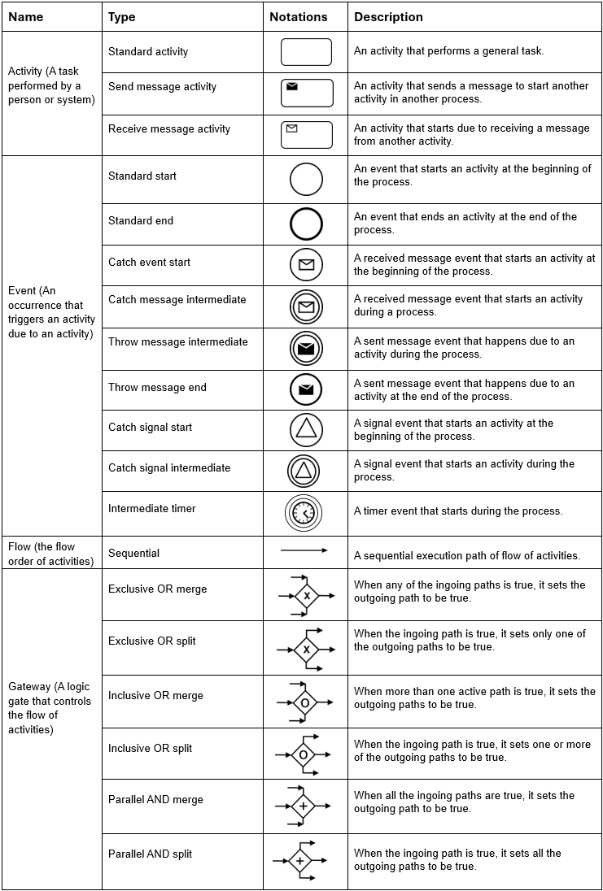

Supplement: Supplementary file 1 [file Image1.jpg]
